# Informing Alzheimer’s Biomarker Communication: Concerns and Understanding of Cognitively Unimpaired Adults During Amyloid Results Disclosure

**DOI:** 10.14283/jpad.2024.151

**Published:** 2024-08-07

**Authors:** Fred B. Ketchum, C. M. Erickson, K. E. Basche, N. A. Chin, M. L. Eveler, C. E. Conway, D. M. Coughlin, L. R. Clark

**Affiliations:** 1 Department of Neurology, School of Medicine and Public Health, University of Wisconsin, Madison, 1685 Highland Avenue, Madison, WI 53705 USA; 2 Department of Medical Ethics and Health Policy, University of Pennsylvania Perelman School of Medicine, Philadelphia, PA 19104 USA; 3 Division of Geriatrics & Gerontology, Department of Medicine, University of Wisconsin-Madison School of Medicine and Public Health, Madison, WI 53705 USA; 4 Department of Mental Health Research, William S. Middleton Memorial Veterans Hospital, Madison, USA; 5 Geriatric Research Education and Clinical Center, William S. Middleton Memorial Veterans Hospital, Madison, WI 53705 USA

**Keywords:** Alzheimer’s Disease, AD biomarkers, biomarker disclosure, dementia education, preclinical AD, ethics and communication

## Abstract

**Background:**

Biomarker results are increasingly disclosed in research and clinical settings, but less is known about how individuals interpret their results or concerns raised during the disclosure visit that may need to be addressed by clinicians to ensure appropriate disclosure.

**Methods:**

Fifty-two cognitively unimpaired older adults aged 65 to 89 years old from the Wisconsin Registry for Alzheimer’s Prevention, who had undergone an amyloid PET scan in the previous 18 months, were enrolled in the disclosure substudy. After ensuring psychological readiness, trained study clinicians disclosed amyloid PET results using a structured protocol. We assessed participants’ level of understanding, concerns, and the perceived personal significance of their biomarker results during the disclosure visit through a series of question prompts in real-time.

**Results:**

Thirty-four received a non-elevated amyloid result and 18 received an elevated result. The average age was 72.2 years (range 65–81); most were women (64%) and non-Hispanic White (92%). Participants understood their results (98%), and both non-elevated and elevated groups provided similar responses around topics of sharing with others, privacy, accuracy of testing, and risk. Participants with elevated results were significantly more likely than those with non-elevated results to want to change their lifestyle (78% vs 12%, p=<0.01) and have questions about their results (61% vs 30%, p=0.05). Participants interpreted the personal significance of results in terms of several themes relating to individual risk status, emotional impact, whether the result was expected, and prevention/planning.

**Conclusion:**

Results show that participants understand their biomarker results, and have a number of concerns during the disclosure process that clinical and research protocols could address. en These findings could be important considerations as effective processes are developed for widespread biomarker disclosure in clinical and research settings.

## Introduction

Alzheimer’s Disease (AD) represents a substantial public health burden, with over 6 million individuals currently affected (1). Biomarkers of AD, beta-amyloid (Aβ) plaques and neurofibrillary deposits of tau can be detected with positron emission tomography (PET) and cerebrospinal fluid (CSF) analysis. These biomarkers can provide information about whether cognitive symptoms are due to AD pathological changes, and can provide risk stratification of the likelihood of converting from mild cognitive impairment (MCI) to dementia (2). Further, these biomarkers may be useful for determining treatment effectiveness, particularly for novel anti-amyloid monoclonal antibody (MAB) therapies (3). However, AD biomarker testing has had limited uptake in clinical care, partially due to specialty resources required to conduct the tests. Rapid progress towards accurate and non-invasive blood tests for AD biomarkers will improve testing accessibility, and novel (MAB) therapies will require disclosure to make biomarker information medically actionable. Currently MABs are approved for Mild cognitive impairment (MCI) and mild dementia, though clinical trials (AHEAD and TRAILBLAZER-ALZ 3) in asymptomatic patients at risk for AD are underway (3). The combination of approvals for MAB therapies and blood-based AD biomarker tests is quickly ushering in a future that will likely include more opportunities for testing and disclosure of biomarkers for AD and other neurodegenerative diseases, particularly for individuals with few or no symptoms.

Prior studies on disclosure of AD biomarkers highlight three primary goals: minimize psychological distress; ensure individuals are informed about their results; and enable individuals to act on these results (4–6). Several guidelines for disclosing results have been developed. Steps for disclosure include determining whether disclosure is appropriate; providing pre-test education and obtaining consent; performing testing and returning results; and follow-up (4, 5, 7). While there have been questions about the potential psychological impact of disclosure (8), data show that disclosure is generally safe (9, 10) and that pre-disclosure education can be effective (11). Limited evidence indicates that individuals generally understand and can recall the meaning of their results, though these data have typically been collected several weeks after disclosure (12, 13). More data are needed from the timepoint of disclosure itself, to evaluate if individuals adequately understand their biomarker results, and to characterize concerns about their results during the disclosure process.

We developed a protocol to communicate biomarker results and tested it in an observational cohort of cognitively unimpaired individuals, previously showing that it was feasible for study clinicians, safe, and participants felt generally satisfied with the visit (14). This study aimed to assess the understanding of results and concerns of individuals during the amyloid PET disclosure process.

## Methods

### Study design

**Figure 1:**
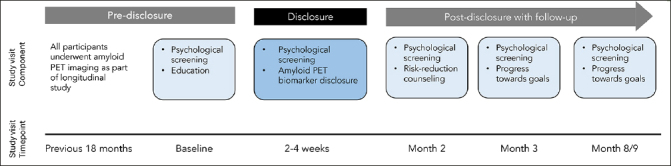
Overview of study visits

The study design included three visits consisting of an education session, disclosure of amyloid PET scan results, and care planning session focused on reducing modifiable risk factors for dementia; as well as three follow-up phone calls to assess additional research outcomes of interest (See Figure 1 for an overview of study visits). In total, study duration was about 9 months. Screening measures for depression (Patient Health Questionnaire-9; PHQ-9), anxiety (Geriatric Anxiety Scale-10 item form; GSA-10), or suicidality (Columbia-Suicide Severity Rating Scale Screen Version; CSSRS) were completed at the baseline visit, and participants were excluded from study participation if thresholds were exceeded (e.g., PHQ-9 > 9, GAS-10 > 9, endorsing suicidal ideation within past month) or they endorsed history of a suicide attempt. Participants were screened using these tools throughout the study, and if thresholds were exceeded, a study clinician followed up with the participant to check-in and conduct an additional risk assessment. We have previously described the disclosure protocol, finding low levels of depression, anxiety, and suicidality among all study participants as well as high levels of protocol adherence and satisfaction among participants and study clinicians (15), and have reported on the effectiveness of pre-disclosure education (11). In the present manuscript, we report results of a cross-sectional study of the knowledge and concerns of cognitively unimpaired adults about receiving their amyloid biomarker results during the disclosure visit.

### Standard Protocol Approvals, Registrations, and Patient Consents

The University of Wisconsin-Madison Institutional Review Board approved the study protocol, and all participants provided written informed consent to participate in the study (IRB #2019-1408).

### Setting and Participants

We recruited participants from the Wisconsin Registry for Alzheimer’s Prevention (WRAP), a longitudinal observational research cohort of largely cognitively unimpaired adults who enrolled in late-midlife and have been followed over time (16). The cohort is enriched for parental family history of Alzheimer’s disease clinical syndrome. A subset of the WRAP cohort had completed an amyloid PET scan within 18 months of disclosure study enrollment. From within this subset, we sought participants who were aged between 65 and 89 years of age, cognitively unimpaired (no diagnosis of mild cognitive impairment or dementia), and no active DSM-5 disorder (including active major mood disorders, psychotic disorders, alcohol/substance use disorder within the past year, history of bipolar I or schizophrenia spectrum disorders). We excluded participants if screening indicated moderate to severe depression or anxiety, suicidal ideation within the past month, or history of suicide attempt.

To assess amyloid burden, participants completed a dynamic Pittsburgh compound B (PiB) PET scan, rated on a 4-point scale by three experienced raters (17). For the purposes of disclosure, the only rating that was considered amyloid elevated was a rating of 3. Ratings of 2, 1 or 0 were disclosed as ‘not elevated’.

We based our disclosure process on best practices in the AD literature (5, 7, 18), and refined the process in consultation with local experts in clinical AD, biomarker imaging, neurology, geriatrics, and neuropsychology. All disclosure visits were conducted over televideo (65% with participants in a remote location, 35% with participants onsite in a separate room) by a member of the study team who had been trained using a combination of didactics and direct observation by a clinician (NC) to use the protocol outlining the disclosure process (available upon request). No amyloid results were disclosed to WRAP participants outside of this disclosure protocol.

During a pilot test phase we provided study clinicians with a script for communicating results. After preliminary evaluation, the script was modified to incorporate topical prompts, to ensure uniform discussion of relevant information during disclosure. Here, we report on the 52 participants who underwent disclosure using the modified protocol. Visits took place from March 24, 2021 until March 11, 2022.

### Data Collection

**Figure 2:**
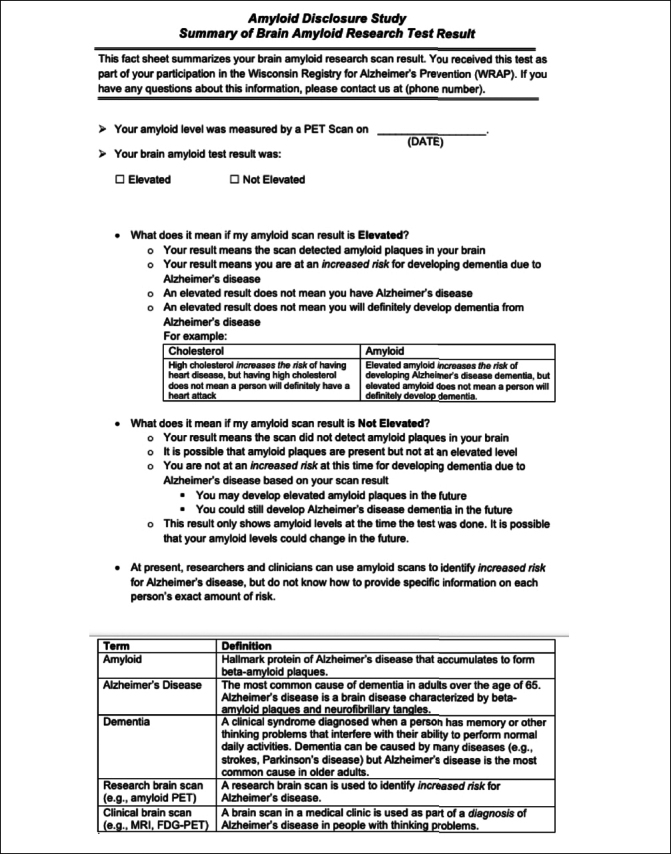
Sample Amyloid Results Disclosure Sheet

Before sharing results during the disclosure visit, participants were screened, and study team members assessed whether disclosure was appropriate by asking participants about any new psychosocial stressors or mood symptoms using the question “Have you experienced any particularly significant or stressful events in your life since your last visit with us?”. If the answer was yes, study team members made a clinical determination about whether disclosure was appropriate at that visit. The study team also confirmed that participants still wanted to learn their results. Next, study clinicians gave participants a result sheet showing their amyloid PET levels as “elevated” or “not elevated” and reading off standardized descriptions of the meaning of results (Figure 2).

After communicating results, study clinicians confirmed participants’ understanding by asking “Tell me what your scan result showed”. We elicited participants’ initial reactions to disclosure by asking one open-ended question, “what is the significance of these results to you?” Study clinicians then followed-up to identify any unaddressed questions participants had by asking: “Do you have any questions about your result?” and “Do you have any questions about your risk of having Alzheimer’s disease?” Responses were recorded by the researchers rather than collected via survey to ensure that study clinicians could provide real-time feedback to participant questions.

After these open-ended questions, study clinicians followed-up with several closed-ended question prompts to assess participant concerns. We based questions on a review of participant concerns in the disclosure literature (19–21), asking: Will you share your result with family or friends? Do you have any concerns about the accuracy of your result? Do you have any concerns about discrimination or privacy? Will your result change any of your behavior? Team members recorded participants’ “yes/no” answers to prompts.

Study team members also noted whether any participants appeared to have a strong emotional reaction during the disclosure visit. This was a clinical determination by team members conducting the visit, all of whom had prior experience in a clinical field (e.g. medicine, neuropsychology).

### Data Analysis

Statistical analysis and generation of figures were carried out using R Statistical Software (v4.2.1) (22). Descriptive statistics were generated to summarize participant demographics. We used descriptive statistics to measure participant responses to each of the six question prompts about participant concerns. We assessed participant understanding by classifying participant answers to the question about what their scan showed as “correct/incorrect”. Acceptable answers referenced the amyloid result, while statements that did not mention amyloid were incorrect. Chi-square analyses and distributions were evaluated to compare participants with an elevated and non-elevated amyloid result.

Responses to the open-ended questions were recorded as verbatim as possible by the disclosing study team member, and were coded thematically using qualitative content analysis (23). We used a team-based, inductive and deductive approach to identify key themes (24). Team members were chosen based on their expertise with qualitative research and/or AD disclosure research. Team members read responses to develop a coding scheme, which was refined until thematic saturation was reached. Each response was reviewed by team members to assign codes, and each response could be coded with multiple themes. Discrepancies were resolved through team discussion.

### Data Availability

Anonymized data will be shared by reasonable request from any qualified investigator.

## Results

### Sample characteristics

52 cognitively unimpaired older adult participants completed the disclosure visit with the amended protocol, of whom 34 had non-elevated and 18 had elevated amyloid results. Table 1 shows participant demographics.

**Table 1:** Participant characteristics

	**Overall**	**Non-elevated amyloid**	**Elevated amyloid**
Number of participants	52	34	18
Age, mean (range)	72.2 (65–81)	72.8 (65–81)	71.0 (65–79)
Gender			
Women, N (%)	33 (64)	24 (71)	9 (50)
Men, N (%)	19 (36)	10 (29)	9 (50)
Race			
Non-Hispanic White, N (%)	48 (92)	32 (94)	16 (89)
Non-Hispanic Black/African-American, N (%)	3 (6)	1 (3)	2 (11)
Asian, N (%)	1 (2)	1 (3)	0 (0)
Education, > Bachelors degree, N (%)	36 (69)	23 (68)	13 (72)
Family history of dementia, N (%)	33 (64)	19 (56)	14 (78)
Duration in WRAP study, mean (range in years)	17.1 (4–21)	17.0 (4–21)	17.2 (4–21)
Average number of PET scans, mean (range)	3.0 (1–6)	2.8 (1–5)	3.4 (1–6)

### Study clinician assessment of knowledge during disclosure

To ensure that participants had an accurate understanding of their results, study clinicians assessed participant knowledge by asking them to state in their own words what their scan showed. Out of 52 participants, 51 (98%) were able to accurately convey whether their scan showed “not elevated” or “elevated” levels of amyloid plaque (100% vs 94% of participants, respectively, *χ*^2^=0.11, p=0.74).

### Participant concerns during disclosure

**Figure 3:**
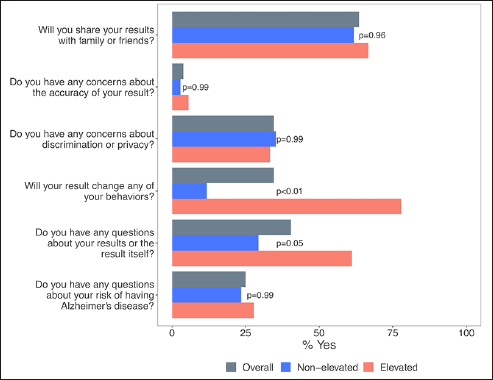
Participant concerns during amyloid PET disclosure visit *Proportion of respondents indicating “yes” to a question prompt. Overall (N=52), non-elevated (N=34), elevated (N=18). P-values calculated from chi-square tests.

Participants with non-elevated and elevated amyloid results expressed similar perspectives during the disclosure visit for several questions: among all participants, 62% of those with non-elevated and 67% of those with elevated results (*χ*2=0.00, p=0.96) planned on sharing result with family or friends. Almost all (97% with non-elevated and 94% with elevated (*χ*2=0.00, p=0.99) had high levels of confidence in testing, and most were not concerned about discrimination or privacy (65% with non-elevated and 67 with elevated (*χ*2=0.00, p=0.99)). Participants with elevated results were more likely to want to change their behavior to reduce risk compared to those with non-elevated results (78% vs 12%, *χ*2=22.72, p=<0.01). Those with elevated results were also more likely to have more initial questions about their results (61% vs 30%, *χ*2=3.68, p=0.05). Frequently asked questions focused on prognosis, the meaning of results, and plans for further testing. Family history of dementia, which was not prompted, was additionally mentioned by 11 participants (21%), five of whom had non-elevated and six of whom had elevated results. Concerns are listed in Figure 3.

### Personal significance of results for participants

Participants mentioned four themes in response to the open-ended question, “what do the results mean to you?”, shown in Table 2. The most frequently mentioned theme, cited 71% of the time, was related to (1) individual risk status. This included comments in which participants summarized their result in terms of their risk for AD, and/or commented that they might still develop AD (for those with a non-elevated result) or might not actually develop AD (for those with an elevated result). The (2) emotional impact of the result was mentioned by 27% of participants. Participants with not elevated results described a sense of relief, while those with elevated results described worry and uncertainty. When sharing emotions, several participants acknowledged that their amyloid result did not determine whether they would develop AD, but was a snapshot of their present condition and might be different in the future (e.g. those with elevated amyloid would not necessarily develop dementia due to AD; and those with non-elevated amyloid could still develop dementia due to AD). No participants had strong emotional reactions, as judged by study clinicians. The theme mentioned 14% of the time was that (3) participants expected their result, either because they had a family history (for participants with elevated amyloid) or because they felt asymptomatic (for participants with non-elevated amyloid). Finally, 10% of responses indicated that (4) participants thought the result would impact their behavior through motivating planning or prevention.

**Table 2:** Themes indicating the personal significance of results

**Theme**	**Overall** number of participants who mentioned the theme (as proportion of total participants, could mention multiple themes)	**Non-elevated amyloid** participants who mentioned the theme (as proportion of all participants who mentioned specific theme, could mention multiple themes)	**Elevated amyloid** participants mentioning the theme (as proportion of all participants who mentioned specific theme, could mention multiple themes)
Individual risk status, N (%)	38/52 (73)	26/38 (68)	12/38 (32)
Emotional impact of the result, N (%)	16/52 (31)	14/16 (88)	2/16 (12)
The result was expected, N (%)	7/52 (13)	3/7 (43)	4/7 (57)
Prevention and planning, N (%)	7/52 (13)	1/7 (14)	6/7 (86)

## Discussion

Given the potential impacts of AD biomarker disclosure and the opportunity to positively impact brain health (25), there is a need to assess the effectiveness of existing guidelines and improve disclosure processes for clinicians and researchers in multiple settings with diverse populations. Using a previously described protocol incorporating recommended practices for AD biomarker disclosure (11, 15) we assessed the understanding and concerns of cognitively unimpaired individuals in a longitudinal observational cohort immediately after they learned their amyloid PET results. Participants with elevated and non-elevated amyloid expressed several similar concerns, but differed in that the former were more likely to want to change their behavior to reduce their risk for developing dementia and had more questions about their results. To our knowledge, this is the first study to capture the concerns of individuals learning their risk for AD during the disclosure visit itself, and we identify opportunities to inform and enhance the disclosure process.

A key goal of biomarker disclosure is ensuring that individuals understand their results, and prior qualitative studies have shown that individuals have generally understood the meaning of their biomarker results when assessed several weeks after disclosure (12, 13). We assessed participant understanding of results during the disclosure encounter, modeling a practical, real-time assessment of understanding and allowing for study clinicians to give participants feedback and additional education if needed. We used structured patient communication utilizing strategies like the ‘teach-back’ method, which has been shown to improve patient education (26). This direct feedback loop also provides opportunities for clinicians to adapt their pre-test education to their specific clinical population (27). However, this real-time assessment may require training for providers to ensure they have a thorough understanding of biomarkers and can adequately assess patient knowledge.

Limited data exist about what individuals wish to know about their biomarker results. In a single previous qualitative study in a secondary prevention trial, all participants desired more quantitative information about the amount of amyloid detected, which was more pronounced among those with ‘elevated’ amyloid PET scan results (13). In our study, all participants were given the opportunity to ask questions after they received their PET results. Individuals were aware of the limitations of biomarker results, as snapshots of current levels of risk that could be different in the future. In line with previous work, more participants with elevated amyloid wanted information about the meaning of their result compared to those with non-elevated amyloid. Nonetheless, when all participants were next asked whether they had any follow-up questions about their risk for AD, a similar proportion in both groups had questions. Similarly, regardless of amyloid result the majority of participants answered open-ended question about what the results meant to them by referring to their personal risk levels.

These data suggest that individuals enrolled in observational research studies have a strong desire to understand their personal risk and its implications for their brain health, highlighting a pressing need to provide meaningful and actionable information during AD biomarker disclosure (6). While our work focused on cognitively unimpaired older adults, data from memory clinic consultations suggests that individuals with cognitive symptoms also desire to learn practical information, which may include strategies to improve brain health (28). Indeed, the timepoint of disclosure may present a unique opportunity to equip individuals at risk for AD to positively impact their behavior: in our study, significantly more participants with elevated than with non-elevated amyloid planned to change their lifestyle. This is similar to other data showing that cognitively unimpaired adults with elevated amyloid were more likely to report behavior changes after learning their results (13).

According to the Health Belief Model (29), a widely used theoretical model to explain health behaviors that has been successfully used in health interventions such as stroke and breast cancer screening (30, 31), several factors influence health behaviors. Individuals are more likely to change their behavior if they perceive themselves as “susceptible” to a health risk; and behavior is influenced both by the perceived severity of the illness and perceived benefits of various courses of action to address their risk (29). While acknowledging that health behaviors are extensively influenced by social context and structural factors (32, 33), learning biomarker results may be an especially opportune time to initiate behavioral change for individuals at risk for AD.

Using best practices for risk communication, clinicians can share recommended strategies to improve brain health tailored to individuals’ personal risk profile (e.g. management of blood pressure or obesity) (25, 34). In the future, this may be paired with recommendations for disease-modifying treatment as well as part of a personalized treatment plan (35). Specific risk information can also facilitate long-term planning, allowing individuals to prepare their personal, legal, and financial affairs (36, 37). In cases where individual risk cannot be reliably estimated, strategies to provide information may include providing ranges of time to onset of cognitive symptoms rather than precise estimates, or describe outcomes in terms of qualitative scenarios and their impact for the patient and family (27, 38).

Disclosure of biomarker test results has consistently been found to be associated with only transient increases in psychological distress in cognitively unimpaired adults (10). Most of these data are from standardized psychological assessments, though some limited qualitative data exist: a qualitative sub-study in a preclinical AD trial suggested that participants with non-elevated amyloid levels had a positive outlook on the future, while those with elevated levels had a mix of positive and negative outlooks (13). In that same cohort, participants in both groups also typically expected the results they received, for instance because of their family history (12); and 1 out of 5 participants would consider physician assisted death (PAD) if they became cognitively impaired, were suffering, or a burden to others (39). Finally, a qualitative study of reactions to a “negative” amyloid result obtained as part of the screening process for a preclinical AD trial in a different cohort showed that all participants expressed relief at their results (40).

These data clarify the psychological impact of learning biomarker results in the short term but do not reflect the experiences of patients and questions clinicians may face during the disclosure process itself. Participants in our study indicated that a negative result provided a sense of relief, while there was some worry or uncertainty about positive results. Clinicians did not observe strong emotional reactions or significant distress, and no participants reported plans for self-harm (15). These findings are in line with what has typically been reported several weeks after disclosure in the literature. Participant responses may reflect that compared to general populations our cohort was highly knowledgeable about AD and often had personal experiences with the disease, and were thus better prepared for an elevated test result. This raises the possibility that there are benefits to education prior to disclosure, which may even be more important prior to implementing widespread disclosure in clinical settings. Clinicians need to be trained in delivering distressing news, and various evidence-based strategies are available. (41, 42).

Our study contributes initial evidence on the perspectives of individuals learning their AD risk about sharing results and discrimination/privacy. Limited data show that research participants are interested in sharing their biomarker results with support persons, such as family or friends (36). In our study, roughly two-thirds of participants wanted to share their results, regardless of amyloid result. This may be because participants with normal amyloid levels wanted to share a sense of relief with others, while those with elevated levels wanted to share information about their risk. To respect individuals’ desires to share their results, disclosure protocols could incorporate mechanisms communicating the significant of biomarker results with the “dyad” of participant and support person(s) (37). Having disclosure protocols that consider support persons in clinical practice will likely be even more important, as family members often play an essential role in the care of older adults, particularly those with cognitive complaints.

Several participants with both non-elevated and elevated amyloid levels were concerned about discrimination or privacy if their results were shared with others, including their doctors or health insurance. We were not able to collect follow-up data to further characterize these perspectives, but it is possible that individuals without elevated amyloid worried about discrimination because someone might learn they were tested for AD. Arguably the concern about discrimination among individuals even without symptoms suggests that these participants had a strong sense of the stigma surrounding AD (43, 44). There are currently only limited legal protections for individuals with preclinical AD, exposing these individuals to workplace or insurance discrimination (45). The level of concern about participants supports ongoing education about this issue prior to disclosure, as recommended by some guidelines (4), and indicates more work is needed in this area.

This study has several limitations. First, our sample was drawn from a research population that is not representative. Our participants were mostly non-Hispanic White, highly educated, had a family history of AD, and had already been involved in AD research for several years. Because of high levels of pre-existing knowledge, they may have had different concerns and experiences from more general populations. Enrollment of cohorts into AD research has been influenced by recruitment strategies (46), and it will be essential for future studies to enroll participants from multiple racially and ethnically minoritized groups. This may also entail that clinicians must use different approaches to communicate results and assess understanding. Second, participants did not have significant preexisting mental illness, and we assessed participant distress prior to disclosure. This may not be representative of broader, clinical populations, in which there may be higher rates of mental health diagnoses and anxiety about results may lead to participants expressing other concerns. Finally, a trained clinician disclosed results, but given the legal requirement to immediately make test results available in the electronic medical record (EMR), this may not reflect future disclosure practices in which individuals may learn results outside of a disclosure visit. Streamlined processes for follow-up scheduling and counseling are needed to ensure patients receive this sensitive information with the appropriate context and guidance on next steps (47).

This is the first study to provide evidence about the experiences and concerns of cognitively unimpaired individuals receiving information about their risk for AD during the disclosure process itself, through real-time feedback from participants obtained by study clinicians. In the future, many more individuals will likely learn their risk for AD in both specialist and primary care settings, a development that may be accelerated by the broader availability of non-invasive blood biomarker testing. Effective and ethically appropriate clinical disclosure practices must not only include the various components recommended by existing guidelines, but must also integrate these elements into a process to achieve the goals of biomarker disclosure, so that individuals: 1) are fully informed about their results; 2) are less likely to experience significant psychological distress and have support available if distress does occur; and 3) are empowered to act on these results. The emphasis on each element in this process may vary based on the population learning results: ethical disclosure practices attempt to maximize the potential benefits of disclosure (e.g. access treatment or clinical trials, ability to plan, make positive health changes, or know more about personal health status) while minimizing potential harms (e.g. unclear clinical utility, limited prognostic value, or negative psychosocial outcomes).[48, 49] While individuals with mild symptoms may have access to treatments, there are currently no approved or effective treatments for asymptomatic individuals, meaning there may be fewer obvious benefits to testing and disclosure (48). This suggests that researchers or clinicians disclosing to asymptomatic individuals should more carefully consider how to maximize benefits by enabling individuals to act on results, at the same time as they minimize possible negative psychological reactions.

We have previously shown that results can feasibly be delivered in a virtual format, and that a brief pre-disclosure intervention is efficacious in increasing knowledge among participants. The present study provides evidence that a structured disclosure process can lead to adequate understanding of results and address the concerns of those learning their AD biomarkers. This study also highlights areas about which additional evidence is needed to further optimize disclosure processes, including education and communication prior to testing, clear strategies to address individual risk, and suggestions for behavioral change and medical-legal planning. To identify the best approaches to biomarker results communication to support varied research and clinical populations, future research should focus on clinically representative populations as the disclosure of AD risk becomes more frequent.

## Notes

*Consent Statement:* The University of Wisconsin-Madison Institutional Review Board approved the study protocol, and written informed consent was obtained from all participants in this study.
